# In vitro resistance development gives insights into molecular resistance mechanisms against cefiderocol

**DOI:** 10.1038/s41429-024-00762-y

**Published:** 2024-07-30

**Authors:** Richard Kriz, Kathrin Spettel, Alina Pichler, Katharina Schefberger, Maria Sanz-Codina, Felix Lötsch, Nicole Harrison, Birgit Willinger, Markus Zeitlinger, Heinz Burgmann, Heimo Lagler

**Affiliations:** 1 Division of Infectious Diseases and Tropical Medicine, Department of Medicine I, Medical University of Vienna, Vienna, Austria; 2 Section Biomedical Science, Health Sciences, FH Campus Wien University of Applied Sciences, Vienna, Austria; 3 Division of Clinical Microbiology, Department of Laboratory Medicine, Medical University of Vienna, Vienna, Austria; 4 Department of Clinical Pharmacology, Medical University of Vienna, Vienna, Austria; 5 Present Address: Pediatric Laboratory, Department of Laboratory Medicine, Medical University of Vienna, Vienna, Austria

**Keywords:** Microbial genetics, Antimicrobial resistance, Antibiotics

## Abstract

Cefiderocol, a novel siderophore cephalosporin, demonstrates promising in vitro activity against multidrug-resistant Gram-negative bacteria, including carbapenemase-producing strains. Nonetheless, only a few reports are available regarding the acquisition of resistance in clinical settings, primarily due to its recent usage. This study aimed to investigate cefiderocol resistance using an in vitro resistance development model to gain insights into the underlying molecular resistance mechanisms. Cefiderocol susceptible reference strains (*Escherichia coli*, *Klebsiella pneumoniae*, *Pseudomonas aeruginosa*) and a clinical *Acinetobacter baumannii* complex isolate were exposed to increasing cefiderocol concentrations using a high-throughput resistance development model. Cefiderocol susceptibility testing was performed using broth microdilution. Whole-genome sequencing was employed to identify newly acquired resistance mutations. Our in vitro resistance development model led to several clones of strains exhibiting cefiderocol resistance, with MIC values 8-fold to 512-fold higher than initial levels. In total, we found 42 different mutations in 26 genes, of which 35 could be described for the first time. Putative loss-of-function mutations were detected in the *envZ*, *tonB*, and *cirA* genes in 13 out of 17 isolates, leading to a decrease in cefiderocol influx. Other potential resistance mechanisms included multidrug efflux pumps (*baeS*, *czcS*, *nalC*), antibiotic-inactivating enzymes (*ampR*, *dacB*), and target mutations in penicillin-binding-protein genes (*mrcB*). This study reveals new insights into underlying molecular resistance mechanisms against cefiderocol. While mutations leading to reduced influx via iron transporters was the most frequent resistance mechanism, we also detected several other novel resistance mutations causing cefiderocol resistance.

## Introduction

The rise of multidrug-resistant (MDR) Gram-negative bacteria (GNB) presents an escalating public health threat resulting in a major global health burden including higher case fatality rates, morbidity, and increasing health expenses [1]. With the emergence of novel resistance patterns and new underlying resistance mechanisms, clinicians and researchers face increasing challenges in the treatment and management of these infections, necessitating the development of new antibiotics and a comprehensive understanding of the resistance mechanisms [2, 3]. According to international organizations, one of the major public health challenges of our time is the emergence of antimicrobial resistance [4, 5].

Cefiderocol is a newly developed catechol-substituted siderophore cephalosporin with a new mode of bacterial cell wall entry utilizing the bacterial iron transport system. Cefiderocol binds to extracellular iron, forming a chelate complex that is transported into the cell. In the periplasmic space, cefiderocol inhibits peptidoglycan synthase by binding to penicillin-binding protein 3 (PBP3), leading to subsequent cell lysis [6]. In addition to the beta-lactam core of cefiderocol, the C-3 side chain is similar to the side chain found in cefepime, which increases water solubility and resists degradation by beta-lactamases. Additionally, a catechol moiety attached to the C-3 side chain allows cefiderocol to form a chelating complex with ferric iron. On the other hand, the C-7 side chain of cefiderocol is the same as in ceftazidime and confers stability against beta-lactamases as well as increases activity against *Pseudomonas aeruginosa* [7]. Cefiderocol has been approved by the FDA in 2019 and by the EMA in 2020 for treatment of GNB infections in adults [8, 9].

As the first siderophore-cephalosporin, cefiderocol has shown potent activity against MDR Enterobacterales species, non-fermenting and carbapenemase-producing bacteria in previous studies [10, 11]. Especially for complicated infections with MDR-GNB including New Delhi metallo-beta-lactamase (NDM) producing Enterobacterales, cefiderocol could offer an effective treatment alternative.

Despite demonstrating promising in vitro efficacy, various resistance mechanisms have already been suggested for the development of cefiderocol resistance. These include reduced influx caused by mutations in siderophore receptors of the iron transport system and mutations in porins, target mutations in PBP genes and the presence of antibiotic-inactivating enzymes. Mutations in siderophore receptors (*piuA, cirA, fiuA*) and other components (*tonB, envZ*) involved in iron uptake have been observed in various species such as *Acinetobacter baumannii*, *Pseudomonas aeruginosa*, *Enterobacter cloacae*, and *Klebsiella pneumoniae* [12–14]. Loss-of-function mutations affecting porins have been identified in various species, such as Enterobacterales (OmpF, OmpD) and *Klebsiella spp*. (OmpK35, OmpK36, and OmpK37). Although porin mutations are associated with a small increase in cefiderocol minimal inhibitory concentration (MIC) (2-4 fold), this increase seems to be not sufficient for cefiderocol resistance [15–17]. Furthermore, a few studies have reported target mutations in the *pbp3* gene associated with cefiderocol resistance [18, 19].

A significant challenge in using clinical isolates for resistance research is that the original susceptible isolate is frequently unavailable following the in vivo development of resistance. When performing whole genome sequencing (WGS) in search of the causal underlying molecular resistance mechanism, a genome of an arbitrary reference isolate is typically used for sequence comparison. Consequently, this comparison often reveals a considerable number of mutations and polymorphisms, frequently reaching into the thousands. This abundance of variants presents a major challenge in unequivocally identifying the mutation or resistance gene responsible for the development of resistance.

To solve this problem, one can use a resistance development model where initially susceptible isolates are exposed to an antimicrobial substance in vitro until resistance development occurs. Subsequently, the sequence of the initial susceptible isolate is compared to that of the newly acquired resistant isolate, and the newly acquired mutations are likely to be associated with the resistance development. One study focusing on a spatiotemporal in vitro resistance development approach of bacterial resistance to antibiotics was the study of Baym et al., which visualized impressively this process on an agar plate, allowing for real-time observation of evolutionary dynamics [20].

We chose a temporal approach for in vitro resistance development, utilizing a high-throughput model in microwell plates. The aim of the study was to induce cefiderocol resistance in vitro in susceptible *A. baumannii* complex*, Escherichia coli, K. pneumoniae* and *P. aeruginosa* strains and analyze the newly acquired resistance mutations to assess the putative underlying resistance mechanism using WGS.

## Methods

### Sampling

In this study we used the cefiderocol susceptible reference strains *E. coli* ATCC 25922, *K. pneumoniae* ATCC 10031, and *P. aeruginosa* ATCC 27853 as well as the clinical *A. baumannii* complex isolate 19-628. To confirm cefiderocol susceptibility antimicrobial susceptibility testing using iron-depleted (Chelex, Sigma-Aldrich, Darmstadt, Germany) and cation-adjusted Müller-Hinton-broth microdilution according to the ISO 20776-1 (2019).

### In vitro antimicrobial resistance development model

**Fig. 1:**
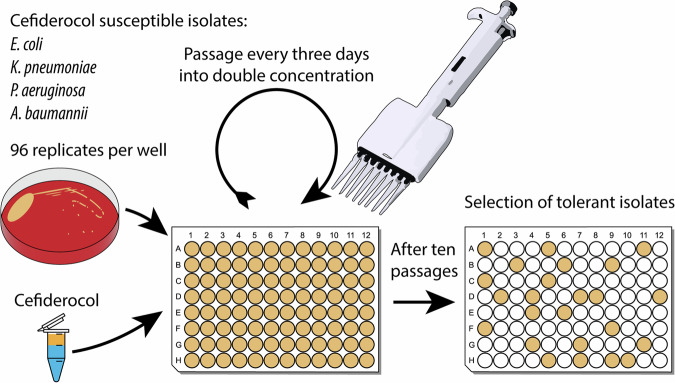
Overview of the methodological workflow of the resistance development model

A high throughput resistance development model was newly designed to induce antimicrobial resistance against cefiderocol in the four susceptible strains listed above. In total 96 replicates of each strain (5 × 10^7^ CFU ml^−1^) were exposed to increasing cefiderocol concentrations in cation-adjusted Mueller-Hinton broth (Becton-Dickinson, Franklin Lakes, USA) in 96-well plates for three days, see Fig. 1. The first batch of resistance development plates had an initial cefiderocol concentration of 0.125 mg l^−1^, and every three days, 100 µl of the suspensions were transferred into a new 96-well plate with the cefiderocol concentration doubled. This cycle was repeated up to 12 times until a maximum cefiderocol concentration of 128–512 mg l^−1^ depending on the species was reached. After resistance development, 5 µl of the suspensions of all wells were streaked on Columbia agar with 5% sheep blood (Becton-Dickinson, Franklin Lakes, USA) to check for growth. The MIC of cefiderocol for all vital isolates was determined using broth microdilution. Resistance to cefiderocol was determined when the MIC exceeded 2 mg l^−1^ according to EUCAST breakpoint. Stable resistance was defined as a persistently elevated MIC observed after discontinuing the selection pressure and five subcultures.

### Whole genome sequencing

We selected the four original cefiderocol susceptible isolates as well as four randomly selected cefiderocol resistance induced clones of *A. baumannii* complex, *E. coli*, and *K. pneumoniae* each, and five cefiderocol resistant clones of *P. aeruginosa* for further investigation with WGS. The DNA of these 21 isolates was extracted based on a phenol-chloroform method. Briefly, several colonies from overnight cultures on Columbia sheep blood 5% agar (Becton-Dickinson, Franklin Lakes, USA) were picked, and the cells were resuspended in 300 µl Tris buffer and 100 µl 5 M NaCl. Then, 400 µl phenol-chloroform-isoamyl alcohol (25:24:1) was added to the mixture. The suspensions were homogenized twice for 15 s at 5000 × *g* in lysing tubes with beads. The supernatant was transferred to a new tube, and 300 µl chloroform was added. The sample was centrifuged at 18,000 × *g* for 5 min, and the supernatant was transferred to a new tube. The last step was repeated, and 225 µl 10 M ammonium acetate was added and vortexed. The tube was then filled to 2 ml with 100% ice-cold ethanol. After centrifugation at 4 °C and 18,000 × *g* for 30 min, the precipitated DNA pellet was washed twice with ice-cold 70% ethanol and resuspended in 50 µl Tris buffer. Following DNA extraction, the Illumina DNA Library Prep kit was used to prepare the samples for WGS according to the manufacturer’s instructions and were sequenced on Illumina MiSeq® Platform on V3 Flow Cell with a final library concentration of 8 pM.

For bioinformatical analysis, the raw reads were quality-trimmed using Trim-Galore 0.4.4_dev [21] and assembled using SPAdes v3.15.4 [22]. The isolates were examined for large deletions and recombination events using Mauve 2.4.0 [23]. The assembled genome of the clinical *A. baumannii* complex strain was annotated using pgap 2023-05-17 [24]. The annotated genomes of the ATCC strains were downloaded from https://genomes.atcc.org/. These genomes were used as a backbone for mapping the resistant isolates using Bowtie2 2.3.4.1 [25]. Variant calling was then performed using samtools 1.16.1 [26] and VarScan v2.4.4 [27], and the variants were annotated using SnpEff 5.0e [28].

## Results and discussion

### Phenotypical changes and resistance profiles

In the in vitro resistance development model, almost all replicates of certain bacterial species continued to grow even when exposed to escalating concentrations of cefiderocol. However, most of these replicates seem to be transiently tolerant cells, as not all of them exhibited a stable resistance. Other temporary resistance mechanisms may enable their survival, including the upregulation of drug efflux pumps or the downregulation of iron transporters, potentially triggered by the gradual increase in cefiderocol concentration.

Following the development of resistance in *P. aeruginosa* using a final cefiderocol concentration of 512 mg l^−1^, 84 out of 96 replicates were viable when cultivated on Columbia agar. Of these 84 isolates, 23 *P. aeruginosa* isolates developed resistance to cefiderocol (Table 1). Among the five sequenced *P. aeruginosa* isolates, the MIC of cefiderocol increased from 0.5 mg l^−1^ to 4–16 mg l^−1^.

**Table 1:** Overview of number of replicates during each step of the study

	*P. aeruginosa*	*A. baumannii*	*E. coli*	*K. pneumoniae*
max cefiderocol concentration [mg l^−1^]	512	512	256	128
Replicates exposed to cefiderocol	96	96	96	96
Replicates viable after exposure	84	31	27	5
Replicates resistant against cefiderocol	23	17	25	5
Replicates sequenced	5	4	4	4

Similarly, after resistance development in *A. baumannii* complex using a final cefiderocol concentration of 512 mg l^−1^, 31 out of 96 wells showed growth when cultivated on Columbia agar. Of these 31 isolates, 17 *A. baumannii* complex isolates exhibited resistance to cefiderocol. Among the four sequenced *A. baumannii* complex isolates, the MIC of cefiderocol increased from ≤0.064 mg l^−1^ to 8– > 32 mg l^−1^.

In the case of *E. coli*, resistance was induced using a final cefiderocol concentration of 256 mg l^−1^, resulting in 27 out of 96 replicates being viable. Among these 27 isolates, 25 *E. coli* isolates demonstrated resistance to cefiderocol. Of the four sequenced *E. coli* isolates, the MIC of cefiderocol increased from 0.125 mg l^−1^ to 16–32 mg l^−1^.

Furthermore, *K. pneumoniae* isolates were subjected to resistance development using a final cefiderocol concentration of 128 mg l^−1^, and five out of 96 replicates were viable. All five of these isolates demonstrated resistance against cefiderocol. Among the four sequenced *K. pneumoniae* isolates, the MIC of cefiderocol increased from ≤0.064 mg l^−1^ to 8–16 mg l^−1^. We selected 17 cefiderocol resistant isolates (four *E. coli, K. pneumoniae* and *A. baumannii* complex and five *P. aeruginosa*) as well as the original corresponding cefiderocol susceptible wild-type isolates for further investigation with WGS.

Many isolates, especially *P. aeruginosa* and *A. baumannii*, showed high tolerability and growth even in high cefiderocol concentrations, although after removing selection pressure the MIC remained at susceptible levels. Therefore, it is important to distinguish between tolerance, persistence, and resistance, which are fundamental survival strategies in bacteria. Tolerance allows bacteria to temporarily endure adverse conditions. Persistence leads to the formation of cells that can resist even high concentrations of antibiotics, although they are practically inert, until the environment changes to more favorable conditions. Resistance involves genetic changes that provide long-term protection against specific stressors. Therefore, it cannot be assumed that all replicates that have grown at high cefiderocol concentrations have automatically acquired stable resistance. Instead, they have developed a transient tolerance and persistence since, in our model, they had the time to gradually adapt to increasing concentrations. An explanation could be changes in gene expression of relevant resistance mechanisms, e.g. overexpression of drug efflux pumps or downregulation of iron transporters. Still, after removing the selection pressure, these isolates quickly reverted to their original susceptible phenotype. On the other hand, nearly all *K. pneumoniae* and *E. coli* isolates that were viable in high cefiderocol concentrations remained cefiderocol resistant even after multiple passaging without cefiderocol exposure, indicating stable mutations in resistance genes.

### Cefiderocol pharmacokinetics and resistance development

Cefiderocol is administered as an intravenous infusion of 2 g every 8 h for 7–14 days, depending on the severity of the infection [29]. Cefiderocol protein binding (PB) has previously been reported as 40–60% [30, 31]. It has also been shown to penetrate various body tissues and fluids, including urine and bronchoalveolar lavage fluid [32, 33]. Understanding these factors is essential for ensuring its effectiveness while minimizing resistance development. This is crucial because suboptimal concentrations provide a fertile ground for resistance development.

Cefiderocol has shown no accumulation and the PK did not change with multiple dosing [34]. The maximum total serum concentration (C_max_) after a cefiderocol 2 g single dose infusion over 3 h was 89.7 mg l^−1^ [35] and over 1 h was 156 mg l^−1^ [34]. Assuming a PB of 40%, this corresponds to a free concentration of 53.82 mg l^−1^ and 93.6 mg l^−1^. Therefore, our in vitro model maximum exposure concentrations (128–512 mg l^−1^) exceeded the C_max_ observed in humans. Additionally, the mutants generated with MIC values ranging from 4 to 32 mg l^−1^ remained below the average C_max_ [36].

It must be noted that our model does not replicate the real in vivo conditions during cefiderocol treatment. In our resistance development model, we employed optimal conditions for resistance development to generate as many resistant isolates as possible to investigate the underlying molecular resistance mechanisms. Therefore, no conclusions can be drawn that cefiderocol would exhibit rapid resistance development in vivo, since our model is not tailored for estimating the in vivo resistance development rate.

### Newly acquired resistance mutations

In this study, all our isolates that acquired cefiderocol resistance consistently showed an increase in MIC against cefiderocol after exposure, with the MIC increasing by 8-fold to 512-fold, see Table 2.

**Table 2:**
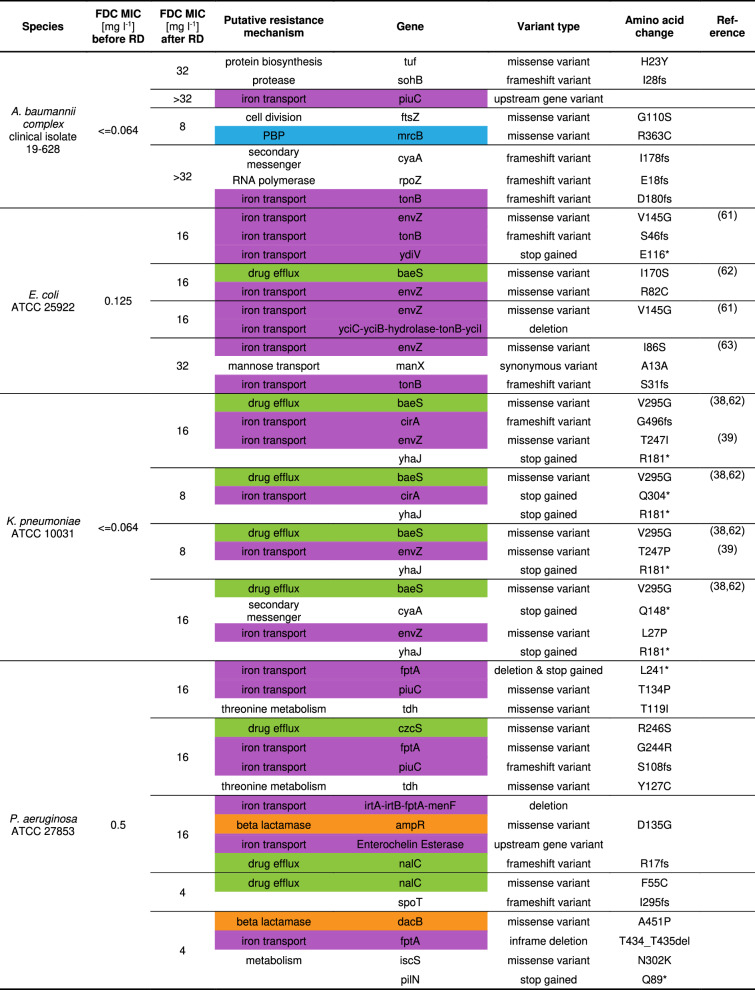
Overview of sequenced isolates, as well as their resistance profile and newly acquired genetic changes

Colors: purple - alteration in iron transport; blue - alteration of penicillin binding protein; green - alteration of drug efflux pumps; orange - alteration in antibiotic inactivating enzymes; FDC - cefiderocol; MIC - minimal inhibitory concentration; RD - resistance development

**Fig. 2:**
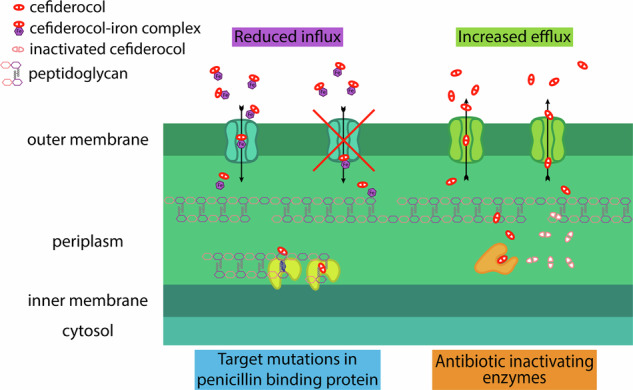
Graphical representation of putative resistance mechanisms associated with cefiderocol resistance

Table 2 and Fig. 2 provide comprehensive data on the MIC of cefiderocol before and after resistance development in the four control strains, as well as the detected putative resistance mechanisms and genetic changes that may be the cause for resistance acquisition to cefiderocol.

Using WGS we identified 42 different mutations within 26 distinct genes. Notably, 35 of these genetic alterations have not been previously described, to the best of our knowledge, representing an expansion of the understanding of resistance to cefiderocol in four different bacterial species.

Of the 17 sequenced resistant isolates, each harbored between one to four newly acquired variants compared to their corresponding wild-type sequences. Notably, the most common variants observed were V295G in *baeS* and R181* in *yhaJ*, present in all four *K. pneumoniae* isolates. The gene with the highest number of variants was *envZ*, exhibiting six different variants across all four *E. coli* and three *K. pneumoniae* isolates.

### Reduced influx of cefiderocol due to impaired iron transport

In our study, we found several newly acquired mutations in genes related to reduced influx. We detected this mechanism 20 times in 13 out of 17 sequenced isolates, making it clear that reduced influx appears to be the predominant resistance mechanism. These mutations were mostly loss-of-function mutations, such as premature stop codons and frameshift mutations, which result in a truncated or highly altered protein and are likely to be associated with a loss of transporter function.

We detected six different mutations (L27P, R82C, I86S, V145G, T247I, T247P) in *envZ*, the sensor protein of the two-component regulatory system OmpR/EnvZ. *EnvZ* may affect cefiderocol uptake as it regulates the expression of genes involved in iron uptake, such as the porins OmpC and OmpF [37]. Mutations in *envZ* have been associated with cefiderocol resistance in in vitro derived isolates in a study [38] and especially mutations at amino acid position T247 seem to have a high impact on the function of the gene [39]. Overall, our findings suggest that mutations in *envZ* play a major role in cefiderocol resistance.

Furthermore, we detected three mutations including two loss-of-function variants, one premature stop codon as well as an inframe deletion (L241*, G244R, T434_T435del) in the gene *fptA* as well as a large deletion of the gene region *irtA-irtB-fptA-menF*. *FptA* is a ton B-dependent receptor for the uptake of the siderophore pyochelin and has already been associated with increased cefiderocol susceptibility when overexpressed [40]. Conversely, in our study, two isolates showed loss-of-function mutations in *fptA*, which likely decreased cefiderocol susceptibility.

We detected a frameshift mutation and a premature stop codon (G496fs, Q304*) in *cirA*, which is a TonB-dependent receptor responsible for the uptake of catecholate siderophores. Recent studies have shown that loss-of-function mutations in *cirA* can lead to decreased uptake of cefiderocol thus leading to resistance, in line with our study [12, 13, 15, 41].

We discovered three frameshift mutations (S46fs, S31fs, D180fs) in the gene *tonB* as well as a large deletion of a genomic region including *tonB* (*yciC-yciB*-hydrolase-*tonB-yciI*), resulting in a loss of protein function. TonB is an inner membrane protein that transduces energy needed for active transport to TonB-dependent receptors in the outer membrane. It is part of the regulating TonB-ExbB-ExbD complex, which plays a crucial role in the uptake of nutrients such as iron [42, 43]. In a previous study, frameshift mutations in *tonB* have already been reported with increased MIC levels to other siderophore-conjugated antibiotics [14].

We identified a premature stop codon (E116*) in the gene *ydiV*. Overexpression of *ydiV* can cause a change in the folding of the protein Fur, impairing its function as an inhibitor of iron receptors, which in turn leads to increased iron uptake [44].

Additionally, we determined a mutation (N302K) in the gene *iscS*, an iron-sulfur protein assembly protein, which is part of a sensing mechanism involved in a wide range of biological processes but the impact on cefiderocol resistance remains unclear [45].

We found two coding mutations as well as an upstream gene variant (T134P, S108fs) in the *piuC* gene, an outer membrane siderophore receptor. Mutations in this gene have previously been linked to resistance against other siderophore-conjugated beta-lactam antibiotics and cefiderocol [46, 47].

Additionally, we detected an upstream gene variant of the gene encoding for enterochelin esterase, which is responsible for the dissociation of the enterochelin iron complex, after entry into the cell [48].

All these mutations in various genes related to iron uptake are likely linked to a reduced influx of cefiderocol, leading to increased resistance to the drug. Therefore, the unique mechanism of cefiderocol to enter the cell seems also to be its vulnerable point, making it the primary target for potential resistance against this antibiotic.

### Antibiotic-inactivating enzymes

Antibiotic-inactivating enzymes are a class of bacterial enzymes that can neutralize the effects of antibiotics by modifying or degrading them. These enzymes are the most common mechanism of antibiotic resistance and can be found in many different types of bacteria.

In our study, we found one mutation (D135G) in *P. aeruginosa* in *ampR*, the transcription factor of the antibiotic-inactivating enzyme *ampC*. This missense mutation is already described in two studies focusing on ceftolozane-tazobactam resistance as well as aztreonam resistance [49, 50] and may lead to a constitutive active gene expression of AmpC.

In our study, a mutation (A451P) in *dacB* gene (also known as PBP4) was found in one *P. aeruginosa* isolate. In a study by Moya et al. it was shown that knockout of *dacB* in *P. aeruginosa* leads to constitutive overexpression of AmpC beta-lactamase as well as activating the CreBC (BlrAB) two-component regulator [51]. This effect was also reported by Ito et al. regarding ceftazidime and cefepime, but not in cefiderocol [52].

Mutations in the beta-lactamase AmpC itself have also been verified as causing cefiderocol resistance by modifying the beta-lactam recognition site which in turn leads to a wider spectrum of degradable beta-lactams. Gomis-Font et al. characterized the mutations L320P, G183D, E247K and T96I, all of which led to an increase in cefiderocol MICs, while having differing effects on other beta-lactams [46]. Another study found deletions in the R2 loop of AmpC, which conferred resistance [53]. However, no mutation in AmpC could be detected in our isolates.

Still, the significance of antibiotic-inactivating enzymes cannot be assessed exhaustively within the scope of this study. The in vitro resistance development model used in this study did not allow for the transmission of genetic elements, such as plasmids, which are often responsible for the transportation of antibiotic-inactivating enzymes. While we found variants, which might be involved in the overexpression of beta-lactamases or lead to increased affinity of beta-lactamases to the antibiotic, it was not possible for the exposed strains to acquire new genes encoding antibiotic-inactivating enzymes via genetic exchange with other bacteria. Thus, the relevance of antibiotic-inactivating enzymes cannot be determined adequately in this study.

### Increased drug efflux

Multidrug efflux pumps are a class of proteins that are present in the cell membrane of bacteria and are involved in the export of a wide range of antibiotics and other toxic compounds from the bacterial cell. These pumps play a significant role in the development of antibiotic resistance by removing the antibiotics from the cell before they can exert their bactericidal effects. In this study, mutations associated with multidrug efflux were found in seven isolates.

The mutation V295G was identified in *baeS*, one part of the two-component regulator *baeSR*, which controls expression of major facilitator superfamily efflux pumps, in all *K. pneumoniae* isolates as well as the amino acid substitution I170S in one *E. coli* isolate. Other studies have previously reported mutations in *baeS* including the V295G mutation in association with increased drug efflux as well as cefiderocol and colistin resistance [38, 54, 55].

Additionally, the mutation (R246S) was located in *czcS* in a *P. aeruginosa* isolate, a part of the two-component regulation system CzcR-CzcS, which regulates the heavy metal drug efflux pump CzcCBA. This drug efflux pump has also been associated with a cross-resistance between zinc and imipenem [56].

Furthermore, we identified mutations (R17fs, F55C) in the gene *nalC* in *P. aeruginosa* isolates, which negatively regulates the multidrug efflux pump MexAB-OprM [57]. As *nalC* downregulates the expression of drug efflux pumps, it is reasonable to assume that loss-of-function mutations can lead to reduced susceptibility. However, the relevance of these mutations in association with cefiderocol resistance remains unclear, as they only occurred in association with other putative resistance mechanisms, which could contribute to the resistance development against cefiderocol.

### Target mutations in penicillin-binding protein

PBPs are transpeptidases responsible for the final stages of peptidoglycan synthesis in GNB and are the target proteins of cefiderocol. Target mutations in the *pbp* genes, leading to structural changes and following reduced binding affinity between antibiotics and the target enzymes, have been implicated in resistance to several beta-lactam antibiotics, including cephalosporins. However, the role of *pbp* target mutations in cefiderocol resistance is not well established. Some studies have suggested that mutations in *pbp3* may contribute to cefiderocol resistance by altering the structure and function of the PBP3 enzyme as it shows the highest affinity for cefiderocol of all PBPs [15, 18, 19]. In our study, a missense mutation (R363C) was found in *mrcB*, which encodes PBP1b in *A. baumannii*. Although PBP1b shows lower affinity to cefiderocol than PBP3 in *A. baumannii* [15], this mutation seems to be most likely causing cefiderocol resistance in the isolate.

### Other unknown mechanisms

We detected a premature stop codon (Q89*) in the gene *pilN*, which is a type 4 fimbrial biogenesis protein [58]. Another frameshift mutation (I295fs) was found in the metabolization gene *spoT* [59]. In the adenylate/guanylate cyclase gene *cyaA* we identified the mutations I178fs and Q148*. Further mutations were found in additional genes like in the elongation factor Tu (H23Y), *ftsZ* (G110S), *manX* (A13A), *rpoZ* (E18fs), *sohB* (I28fs) and *tdh* (T119I, Y127C). In four different *K. pneumoniae* clones, we detected the same premature stop codon (R181*) in the gene *yhaJ*, without any major evidence of a putative resistance mechanism [60]. These mutations, however, always occurred in combination with other mutations potentially explaining cefiderocol resistance.

### Limitations

This is an in vitro study investigating the development of resistance to cefiderocol. Cefiderocol susceptible isolates were exposed to escalating concentrations of cefiderocol, starting with an initial concentration below both the MIC and the therapeutic concentration used in vivo. These in vitro conditions are considered ideal for the development of resistance but are not comparable to an in vivo setting during treatment. Therefore, it cannot be estimated how fast resistance development can occur in vivo during therapy with cefiderocol.

Additionally, the resistance development was conducted in CAMHB rather than in iron-depleted CAMHB. Our aim was to provide optimal conditions for the development of cefiderocol resistance in vitro. Given that our primary objective was to generate as many mutants as possible, this approach proved effective. However, it is possible that some of the resistance mechanisms would be more or less frequent under iron-depleted conditions. Investigating this in a further study would be valuable, as using an iron-depleted medium would better simulate in vivo conditions.

While we have repeatedly subcultured the strains they maintained stable cefiderocol resistance. Still, mutations in iron influx pathways may contribute to a loss of fitness, which may cause them to decline within the microbial ecosystem in vivo or under adverse conditions. Furthermore, the assessment of in vivo pathogenicity becomes a viable consideration, as potential loss of fitness could lead to reduced virulence in the event of infection. In this respect, employing an in vivo model to compare isolates before and after the acquisition of resistance may offer a valuable information regarding pathogenicity.

However, it has been observed that resistance development is possible with improper use of this antibiotic, and many different molecular resistance mechanisms have been identified. Therefore, in vitro resistance development models are very useful tools in identifying underlying molecular resistance mechanisms. Although this model reduces the noise of genetic variants, that must be considered for causing resistance, it is not an exhaustive analysis of the molecular resistance mechanisms. In vitro mutagenesis would be ideal to further analyze the involvement of each variant found in resistance development.

## Conclusion

Our study provides a comprehensive look into the molecular basis of cefiderocol resistance using an in vitro resistance development model. We detected 35 novel resistance mutations putatively conferring resistance to cefiderocol in 26 different genes. These findings, alongside the identification of previously reported mutations associated with cefiderocol resistance, offer a better understanding of the molecular resistance mechanisms and relevant genes involved in cefiderocol resistant isolates.

The most frequent resistance mechanism was reduced influx via iron transporters, but we also detected a wide range of other putative resistance mutations previously not associated with cefiderocol resistance. Even though cefiderocol has demonstrated low resistance rates in clinical environments and exhibits potent activity against MDR-GNB, the potential emergence of resistance is still a looming concern, especially with escalated and inadequately monitored usage of this antimicrobial agent. Therefore, it is crucial to promote considerate use of antibiotics, implement effective surveillance and stewardship programs, and pursue resistance testing to cefiderocol to assess efficacy. As with any antimicrobial agent, adequate and indicated clinical usage is essential to preserve its effectiveness in future clinical settings.

## Data Availability

WGS data is available at https://www.ncbi.nlm.nih.gov Bioproject PRJNA1028938.
